# Evaluation of a Standard Dietary Regimen Combined with Heat-Inactivated *Lactobacillus gasseri* HM1, Lactoferrin-Producing HM1, and Their Sonication-Inactivated Variants in the Management of Metabolic Disorders in an Obesity Mouse Model

**DOI:** 10.3390/foods13071079

**Published:** 2024-04-01

**Authors:** Wei-Chen Shiu, Zhen-Shu Liu, Bo-Yuan Chen, Yu-We Ku, Po-Wen Chen

**Affiliations:** 1 Department of Veterinary Medicine, College of Veterinary Medicine, National Chung Hsing University, Taichung 402202, Taiwan; sponge77625@gmail.com (W.-C.S.); jackynike061548@gmail.com (B.-Y.C.); kyw168@mail.e-land.gov.tw (Y.-W.K.); 2 Department of Safety, Health and Environmental Engineering, Ming Chi University of Technology, New Taipei City 24301, Taiwan; zsliu@mail.mcut.edu.tw; 3 Chronic Diseases and Health Promotion Research Center, Chang Gung University of Science and Technology, Chiayi 61363, Taiwan; 4 Center for Sustainability and Energy Technologies, Chang Gung University, Taoyuan 33302, Taiwan; 5 Animal and Plant Disease Control Center Yilan County, Wujie Township, Yilan County 268015, Taiwan

**Keywords:** anti-inflammation, obesity, metabolic disorder, probiotic, postbiotics, *Lactobacillus gasseri*, high-fat diet, lactoferrin

## Abstract

This study investigated the impact of incorporating various inactivated probiotic formulations, with or without recombinant lactoferrin (LF) expression, into a standard chow diet on metabolic-related disorders in obese mice. After inducing obesity through a 13-week high-fat diet followed by a standard chow diet, mice received daily oral administrations of different probiotics for 6 weeks using the oral gavage approach. These probiotic formulations consisted of a placebo (MRS), heat-inactivated *Lactobacillus gasseri* HM1 (HK-HM1), heat-killed LF-expression HM1 (HK-HM1/LF), sonication-killed HM1 (SK-HM1), and sonication-killed LF-expression HM1 (SK-HM1/LF). The study successfully induced obesity, resulting in worsened glucose tolerance and insulin sensitivity. Interestingly, the regular diet alone improved glucose tolerance, and the addition of inactivated probiotics further enhanced this effect, with SK-HM1/LF demonstrating the most noticeable improvement. However, while regular dietary intervention alone improved insulin sensitivity, probiotic supplementation did not provide additional benefits in this aspect. Inflammation in perirenal and epididymal fat tissues was partially alleviated by the regular diet and further improved by probiotics, particularly by SK-HM1, which showed the most significant reduction. Additionally, HK-HM1 and HK-HM1/LF supplements could contribute to the improvement of serum total triglycerides or total cholesterol, respectively. Overall, incorporating inactivated probiotics into a regular diet may enhance metabolic indices, and recombinant LF may offer potential benefits for improving glucose tolerance.

## 1. Introduction

Obesity denotes a modified health state stemming from irregularities in energy intake, energy balance, gut microbiota, and an inappropriate diet, influenced by genetic and environmental factors. It is operationally characterized as the accumulation of excessive fat, adversely affecting health status [1]. Nowadays, obesity has been characterized as a chronic disease, reaching pandemic proportions and being associated with various metabolic disorders, including type 2 diabetes (T2DM), cardiovascular ailments, and cancer [2,3]. Hence, there lies imperative significance in the treatment or prevention of obesity and metabolic disorder. To date, various interventions, including caloric restriction, aerobic exercise, pharmacological treatments, and bariatric surgery, have shown efficacy in promoting weight loss and mitigating the associated risks of disease in individuals grappling with obesity [3,4,5,6]. However, at both individual and community levels, the prevention and treatment strategies for obesity have not achieved success in the long run [7]. Nonetheless, lifestyle modifications remain the primary therapy for obesity and related metabolic disorders. For instance, calorie restriction (CR) diets have gained recognition for their role in promoting health, extending lifespan, and averting the onset of metabolic and age-related ailments [8]. However, maintaining long-term weight loss has proven to be a formidable challenge, primarily owing to intricate interplays between hormones and behavior [9,10,11]. These interactions frequently culminate in gradual or rapid weight regain following a CR intervention [12,13].

One potentially ideal strategy for treating obesity involves the manipulation of gut microbiota. For example, accumulating scientific evidence supports the role of probiotics, probiotic components, and their metabolites in regulating host lipid, sugar, and cholesterol metabolism, as well as in suppressing oxidative stress and mitigating endoplasmic reticulum stress. Certain probiotics also induce improvements in gut microbiota composition and inhibit chronic low-grade inflammation, thereby preventing and ameliorating obesity and related chronic diseases [14,15,16,17]. The advantages of manipulating gut microbiota or implementing probiotic-related interventions have been thoroughly reviewed and succinctly summarized as follows. First, this therapeutic approach is deemed safe, with no reported adverse effects, and it is well tolerated, making it suitable for long-term use. Second, the modulation of gut microbiota through probiotic treatment or dietary intervention, owing to its beneficial effects, can impact body weight, influence glucose and fat metabolism, enhance insulin sensitivity, and mitigate chronic systemic inflammation [18]. However, a comprehensive literature review reveals that the favorable effects of probiotic supplementation in individuals with obesity are linked to various factors. These include the nature of the probiotic strain, the composition of the probiotic formula (such as single or multiple strains, with or without prebiotics), the duration of the intervention, dosage, and other supplementary activities, such as CR and use of weight loss medications [19].

Probiotics, as defined by the International Scientific Association of Probiotics and Prebiotics (ISAPP), are active micro-organisms, which, when ingested in sufficient quantities, confer health benefits to the host [20]. In recent years, the scientific community has introduced various terms, such as non-viable probiotics, paraprobiotics, ghostbiotics, and notably, postbiotics, to describe inanimate micro-organisms and/or their constituents conferring health benefits [21,22]. Postbiotics are compounds originating from the metabolites or cell wall fragments produced by probiotics. This term encompasses a diverse array of bioactive molecules, such as non-viable or inactivated microbial cells, short-chain fatty acids, vitamins, enzymes, teichoic acid, peptides, and exopolysaccharides, all derived from beneficial micro-organisms [23,24]. Notably, postbiotics have been recognized as safe for use, and documented data highlight the potential of postbiotics and derivatives from probiotics in managing metabolic disorders and associated changes [22,25,26]. Remarkably, the utilization of specific prebiotics and postbiotics has demonstrated efficacy in long-term weight management for the control of obesity [27].

Lactoferrin (LF) is an 80 kDa iron-binding glycoprotein predominantly found in the milk and various exocrine fluids of mammals. It manifests diverse pleiotropic activities, encompassing anti-inflammatory, antimicrobial, antioxidant, immune-regulating, and prebiotic properties [28,29,30]. Additionally, in both low- and high-dose combinations, LF and inulin have demonstrated an additive or supra-additive effect in reducing energy intake, body weight, and adiposity in a diet-induced obese rat model [31]. Furthermore, dietary lactalbumin and LF have also proven effective in enhancing the energy balance, metabolism, and reducing adiposity. Furthermore, the impact of LF appears to be partly independent of caloric intake in a diet-induced obese rat model [32]. To facilitate the concurrent display of functional attributes inherent to both LF and probiotics, we recently developed engineered probiotics proficient in expressing bovine, human, or porcine LF [33]. Subsequently, we further formulated three distinct probiotic supplements, namely lactic acid bacteria (LAB), LAB/LF, and inactivated LAB/LF. The LAB supplement comprised 10 viable LAB without recombinant LF expression; the LAB/LF supplement involved 10 viable LF-expressing probiotics; and the inactivated LAB/LF supplement was derived from 10 inactivated LF-expressing probiotics. Utilizing the NAFLD mice model, the administration of the aforementioned live or inactivated probiotic mixtures derived from host probiotics or LF-expressing probiotics demonstrated a significant and distinct amelioration of hepatic steatosis and inflammation [34].

In light of the observed improvements in metabolic-related disorders following the administration of inactivated probiotics (postbiotics) or LF, we hypothesized that the inactivated singular probiotic strain, specifically *Lactobacillus gasseri* HM1 (HM1), which acts as the host strain for LF-expressing probiotic, may also demonstrate anti-obesity capabilities. Hence, in the present study, we aim to investigate the anti-obesity effects of the inactivated HM1 strain. This comparison extends to evaluating heat-killed and sonication-killed HM1, with a particular emphasis on those expressing LF, representing two distinct formulations of inactivated HM1/LF. Secondly, given the well-known association between dietary control and improved metabolic indices, we also aim to explore the impact of a combination of regular diet (standard chow diet) and supplementation with one of four inactivated probiotic formulations in addressing metabolic disorders.

## 2. Materials and Methods

### 2.1. Probiotic Strain and Postbiotics Preparation

The probiotic strain (*L. gasseri* HM1, our laboratory stock), sourced from human milk [35,36], was cultivated under anaerobic conditions at 37 °C in De Man, Rogosa, and Sharpe (MRS) broth (BD Biosciences, San Jose, CA, USA) for a duration of 24 h. Another *L. gasseri*/BLF strain, the LF-expressing HM1 (HM1/LF), was activated and cultured anaerobically in MRS broth at 37 °C without agitation [33]. The expression of recombinant LF was induced by supplementing cultures of recombinant LF-expressing probiotics with nisin at a concentration of 1 ng/mL (supplemented in fresh medium) for 16 h at 30 °C, as previously described in our report. Moreover, the confirmation of LF expression in probiotics was achieved through SDS page and Western blotting analysis, utilizing an anti-LF antibody [33]. To investigate the varied effects of two probiotic inactivation methods on improving the indicators of metabolic disease, two distinct approaches were employed: high-temperature (heat-killing) and non-high-temperature (sonication-killing) methods were utilized to deactivate the probiotics. Initially, probiotic strains were harvested via centrifugation (Hermle Z 366 K, Wehingen, Germany) at 12,000× *g* for 10 min. The pellets underwent two washes with 10 mL of PBS buffer. Following a cell count, the bacterial concentration was adjusted to 5 × 10^10^ cells/mL using MRS broth. Then, 1 mL of HM1 or HM1/LF probiotics (5 × 10^10^ cells/mL) underwent heat treatment in an autoclave (TM-321, Tomin Autoclave, Taipei, Taiwan) under the following conditions: 121 °C and 1.5 atmospheres of pressure for 15 min. Additionally, another 1 mL of HM1 or HM1/LF probiotics (5 × 10^10^ cells/mL) was subjected to sonication for 5 min at 4 °C using a sonicator (Sonic Material VCX600, Newtown, CT, USA) with a power output of 20 W. Subsequently, the supernatant from both treatments was collected through centrifugation at 15,000× *g* for 5 min at 4 °C to eliminate any precipitates. This procedure yielded four inactivated probiotic formulations, including heat-killed HM1 (HK-HM1), heat-killed LF-expressing HM1 (HK-HM1/LF), sonication-killed HM1 (SK-HM1), and sonication-killed HM1/LF (SK-HM1/LF).

### 2.2. Animal, Experimental Design, and Sample Collection

The animal experiments were conducted with the approval of the Institutional Animal Care and Use Committee of the National Chung Hsing University, under approval number IACUC No. 110-080R. A total of 36 six-week-old male C57BL/6J mice, obtained from the National Laboratory Animal Center in Taiwan, were utilized for this experiment. The mice were housed in a controlled animal facility with an environmental temperature of 26 °C, a relative humidity of 63%, and a 12 h light–dark cycle, providing them with unrestricted access to food and drinking water. After a two-week acclimatization period in this controlled environment, the animals were introduced to the experimental procedures.

To induce an obesity animal model, 30 mice were exposed to a 13-week high-fat diet (HFD), comprising 60% kcal from fat, 20% kcal from protein, and 20% kcal from carbohydrates (D12492; Research Diets, New Brunswick, NJ, USA). Following this 13-week period, the mice were transitioned to a standard diet, specifically the standard chow diet (SCD), for a 6-week duration. The SCD contained 13% kcal from fat, 28% kcal from protein, and 59% kcal from carbohydrates, adhering to the formulation of the 5001 Laboratory Rodent Diet from Lab Diet in the USA. Over the course of these six weeks, five groups of mice underwent daily oral treatments administered via oral gavage, with a dosage of 0.2 mL per mouse: placebo (MRS; Placebo; n = 6), HK-HM1 (n = 6), SK-HM1 (n = 6), HK-HM1/LF (n = 6), and SK-HM1/LF (n = 6). Additionally, another six mice were designated as the normal control group (Normal control), and they were provided with a regular diet for a total of 19 weeks, with the administration of a placebo (MRS) through oral gavage oral ingestion for 6 weeks, starting from the 13th week.

Throughout the experimental period, weekly assessments were conducted to monitor changes in body weight and food consumption. The final weight recorded at the conclusion of the experiment served as the basis for evaluating the effectiveness of weight loss or gain in each group. Following this assessment, the mice were anesthetized, and euthanasia was performed by collecting blood from the heart. The obtained blood samples underwent additional analysis.

Subsequently, liver, epididymal fat, and perirenal fat were harvested from the mice. These tissue samples were preserved in 10% formalin for 24 h, followed by embedding in paraffin and sectioning for histological examination. The tissue sections underwent staining with hematoxylin and eosin (H&E) to observe histopathological changes. The histological analysis employed semi-quantitative criteria in histopathological examination, assessed blindly by a certified veterinary pathologist in Taiwan (CSVP Vet Path No. 0019). Moreover, the degree of lesions was graded from zero to four based on severity: 0 = normal; 1 = slight (<10%); 2 = moderate (10–33%); 3 = moderate/severe (33–66%); 4 = severe/high (66–100%) [37].

### 2.3. Biochemical Analyses

Biochemical analyses were performed on blood specimens collected in BD Vacutainer^®^ SST II Advance blood collection tubes, equipped with coagulation enhancers and separating gels. Following gentle mixing, the samples stood at room temperature for 30 min. Subsequently, they underwent centrifugation at 2000× *g* for 10 min to facilitate serum separation. The resulting serum samples were then forwarded to the National Laboratory Animal Center (NLAC Taiwan) for the quantification of total cholesterol (TC), total triglycerides (TG), low-density lipoprotein cholesterol (LDL-C), high-density lipoprotein cholesterol (HDL-C), and glucose on a 7080 biochemical analyzer (Hitachi, Tokyo, Japan), according to the manufacturer’s instructions.

### 2.4. Testing for Glucose Tolerance and Insulin Sensitivity

We adhered to established experimental protocols, as outlined in prior publications, for assessing glucose and insulin tolerance to evaluate insulin sensitivity in mice before and after a 6-week dietary and probiotic intervention [38]. Glucose tolerance was assessed at weeks 12 and 17 of the experiment, while insulin tolerance was evaluated biweekly during weeks 13 and 18. For the glucose tolerance test (GTT), mice underwent a 6 h fasting period followed by intraperitoneal injections of a 2 g/kg glucose solution. Blood glucose levels were monitored at 0, 15, 30, 60, and 120 min post-injection using a blood glucose monitor (OneTouch, Select Plus; LifeScan, Milpitas, Santa Clara, CA, USA). Regarding the insulin tolerance test (ITT), mice also experienced a 6 h fasting period, followed by intraperitoneal injections of 1 IU/kg insulin. Blood glucose levels were then monitored at 0, 15, 30, 60, and 120 min post-injection using the same blood glucose monitor (OneTouch, Select Plus; LifeScan, Milpitas, Santa Clara, CA, USA).

### 2.5. Statistical Analysis

The results are presented as mean values ± standard deviation (SD). Analysis of variance (ANOVA) was utilized to assess differences between the groups, and Tukey’s post hoc test was employed to evaluate the significance of differences between specific data points. The analysis was performed using SPSS (Version 20.0). Statistical significance was established with a *p*-value of less than 0.05, indicating the presence of meaningful distinctions.

## 3. Results

### 3.1. Weight Fluctuations in Mice throughout the Entire Experimental Period

In this study, our objective was to evaluate the impact of combining a regulated diet with inactivated natural or recombinant LF expression on metabolic indices. Initially, we measured weight variations among mouse groups at the beginning, after a 13-week high-fat diet induction period, and following a 6-week regular diet with four inactivated probiotic supplementations. The body weight values of mice at significant time points during the experiment are presented in Table 1. At week 0, all mouse groups exhibited comparable weights (*p* > 0.05). Subsequently, following a 13-week high-fat diet, including placebo, HK-HM1, SK-HM1, HK-HM1/LF, and SK-HM1/LF, all groups showed significant weight gains, with weights statistically higher than those of Normal control groups (*p* < 0.05). These weights increased by over 42% compared to the normal group, indicating successful induction of obesity in all mouse groups. After an additional 6-week regulated diet with any of the four probiotic formulations (week 19), the weights of the five groups declined considerably, yet remaining higher than those of the normal mouse group. Although the HK-HM1/LF group had a significantly lower weight than the placebo group, the weight gain index did not differ between the placebo and HK-HM1/LF groups. 

**Figure 1:**
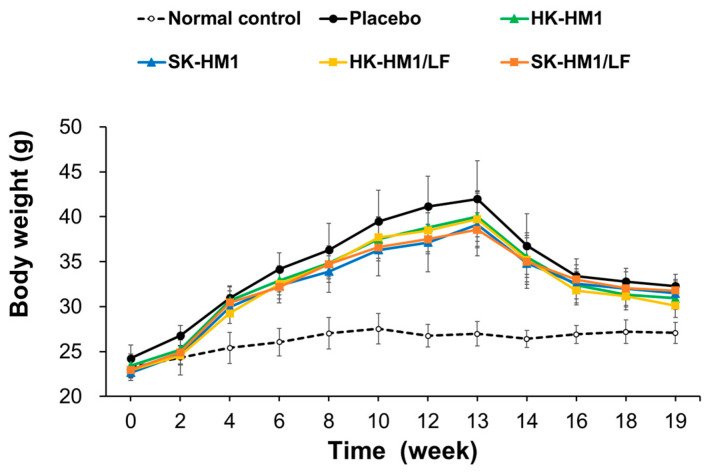
Trends in body weight change curve of mice during the experiment. See Table 1 for the key.

In Figure 1, we further compared the trends in the body weight change curve of mice during the experiment. The results indicate that during the induction of obesity with a high-fat feed, the weight gain trends in the five groups of mice were similar. Additionally, after the regular diet and probiotic intervention, the weight loss trends in the five groups of mice were also similar. Overall, these findings suggest that while a standard chow diet can contribute to weight loss, the administration of any of the four probiotic formulations does not provide additional benefits in reducing body weight. Furthermore, based on the weight reduction curve after 13 weeks, the optimal weight loss effect of dietary control can be maintained for approximately 3 weeks (from week 13 to 16), with the downward trend becoming more moderate after 16 weeks. Nevertheless, we also assessed additional metabolic indices, which could be influenced by the administration of a regular diet or probiotics, as outlined below.

### 3.2. Glucose Tolerance among Treatments

**Figure 2:**
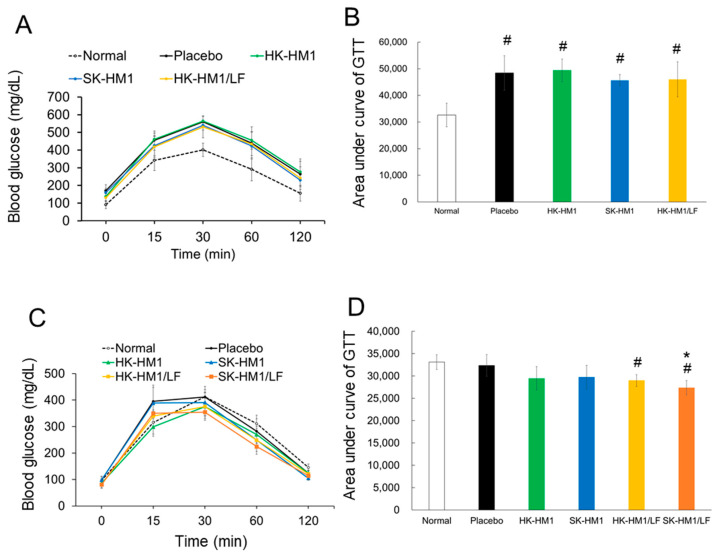
The results of the glucose tolerance test (GTT) in obese mice before and after a 6-week intervention with a combination of probiotics and a conventional diet. (**A**): Variations in blood glucose levels within the obese mice group prior to the intervention of probiotics and a standard diet; (**B**): Total area under the curve computed from A; (**C**): Changes in blood glucose levels within the obese mice group after the intervention of probiotics and a standard diet; (**D**): Total area under the curve calculated from C. ^#^ Statistically significant difference compared to Normal control (*p* < 0.05); * Statistically significant difference compared to placebo + SCD (*p* < 0.05).

The glucose tolerance test (GTT) is a diagnostic tool used to assess glucose metabolism and pancreatic function in the body. It is commonly employed to identify signs of diabetes or abnormalities in pancreatic function. In Figure 2, we conducted GTT evaluations among different groups of mice both before and after a 6-week period of regular diet and probiotic intervention. As described in Table 1 and Figure 1, following a 13-week high-fat diet induction, all five groups of mice (n = 30) were successfully induced into obesity. Given the comparable backgrounds of these obese mice, we specifically analyzed GTT in four mouse groups: placebo, HK-HM1, SK-HM1, and HK-HM1/LF. As expected, mice in the placebo, HK-HM1, SK-HM1, and HK-HM1/LF groups demonstrated consistent patterns in the elevation and decline of GTT variation (Figure 2A). Moreover, the GTT curves for these four groups at the designated time points of testing (15, 30, 60, and 120 min) were all higher compared to those of the normal mouse group. This outcome indicates that the 13-week high-fat diet intervention indeed worsened the GTT in these mice. To validate these observations, we computed the total area under the GTT curve among the mouse groups based on the data presented in Figure 2A. As depicted in Figure 2B, the placebo, HK-HM1, SK-HM1, and HK-HM1/LF groups indeed exhibited a significantly higher area under the GTT curve compared to the Normal control (*p* < 0.05). Additionally, when considering the surface area under the curve in healthy mice as 1, the respective surface areas under the curves for the placebo, HK-LM1, SK-LM1, and HK-LM1/LF groups (all obese mice) were 1.48, 1.52, 1.40, and 1.41. These results further support the notion that obesity indeed exacerbates glucose intolerance in mice by up to 40–50%.

The glucose tolerance of mice treated with a regular diet combined with probiotic formulations for six weeks is depicted in Figure 2C,D. In Figure 2C, HK-HM1, HK-HM1/LF, and SK-HM1/LF exhibited lower blood glucose levels than the Normal control at most tested time points. To validate this, we also calculated the total area under the glucose curve for each mouse group. As shown in Figure 2D, the placebo group demonstrated glucose tolerance similar to that of the normal mice, suggesting that dietary control alone (regular diet) contributes to improved glucose tolerance. Remarkably, the HK-HM1/LF and SK-HM1/LF mouse groups displayed a significantly lower area under the GTT curve compared to the normal mice (*p* < 0.05). Additionally, the SK-HM1/LF mouse groups exhibited a statistically lower area under the GTT curve compared to the placebo mice group (*p* < 0.05). Finally, assigning the area under the GTT curve of normal mice as 1, our analysis indicates that the probiotics HK-HM1, SK-HM1, HK-HM1/LF, and SK-HM1/LF could improve glucose tolerance by approximately 11%, 10.2%, 12.5%, and 17.4%, respectively.

In summary, these findings suggest that supplementation with SK-HM1/LF probiotic formulations leads to additional improvements in glucose tolerance. 

### 3.3. Insulin Sensitivity across Treatments

**Figure 3:**
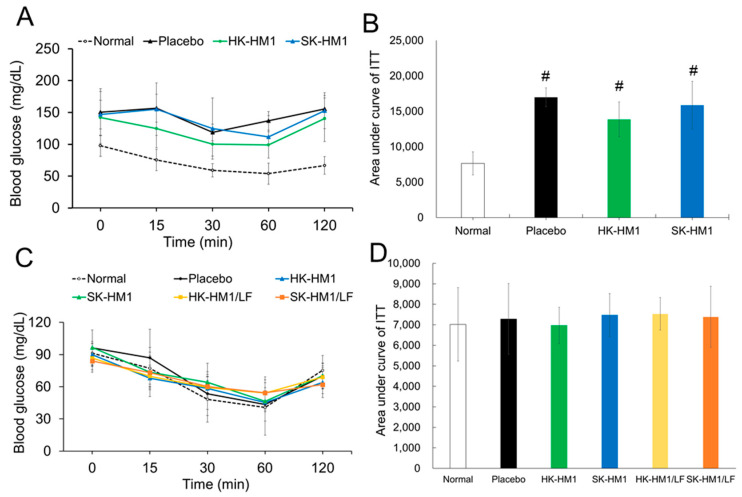
The outcomes of the insulin sensitivity test (ITT) conducted on obese mice both before and after a 6-week intervention involving a combination of probiotics and a conventional diet. (**A**) depicts the fluctuations in blood glucose levels within the obese mice group before the initiation of the probiotics and standard diet intervention. (**B**) represents the total area under the curve computed from the data in A. (**C**) shows the changes in blood glucose levels within the obese mice group following the intervention of probiotics and a standard diet. (**D**) displays the total area under the curve calculated from the data in C. ^#^ Statistically significant difference compared to Normal control (*p* < 0.05).

We also assessed insulin sensitivity before and after a 6-week period of regular dietary intake combined with one of four probiotic formulations (Figure 3). Figure 3A,B depict the ITT results in mice prior to the initiation of dietary and probiotic interventions. Considering that all 30 mice were induced into an obese state (Table 1 and Figure 1), ITTs were conducted on only 18 mice to reveal the baseline insulin sensitivity in these obese mice (Figure 3A,B). In Figure 3A, the glucose response curves for the three obese mice groups (placebo, HK-LM1, and SK-LM1) showed a consistent and significant elevation in blood glucose levels at 15, 30, 60, and 120 min post-glucose administration compared to the healthy control group. Additionally, the glucose concentration area under the curve (AUC) from 0 to 120 min (Figure 3B) demonstrated a significantly higher AUC in all three obese groups compared to the healthy group (*p* < 0.05), with no statistical differences among the obese groups. These results indicate a consistent reduction in insulin sensitivity among obese mice, confirming that obesity indeed worsens insulin sensitivity. Furthermore, normalizing the AUC of healthy mice to 1, the AUC values for the placebo, HK-LM1, and SK-LM1 groups were 1.42, 1.49, and 1.46, respectively. These findings reinforce the notion that obesity exacerbates insulin sensitivity in mice. Comparing these results with Figure 2B, it is evident that obesity similarly deteriorates both insulin sensitivity and glucose tolerance, with both parameters being affected by approximately 40–50%.

Figure 3C illustrates the insulin sensitivity results in obese mice after 6 weeks of dietary control and supplementation with probiotics. The glucose response curves among the six groups exhibit strikingly similar trends, indicating comparable insulin sensitivity across these groups. Further analysis of the overall insulin sensitivity among different groups, measured by the area under the curves (Figure 3D), reveals no statistically significant differences among the six groups of mice. These findings suggest that dietary control alone effectively improves insulin sensitivity in obese mice. However, regardless of the probiotic formulation administered, the mice did not experience additional benefits in insulin sensitivity.

### 3.4. Improvement Benefits of Dietary Control and Oral Probiotics in Tissue Pathological Changes in Obese Mice

**Figure 4:**
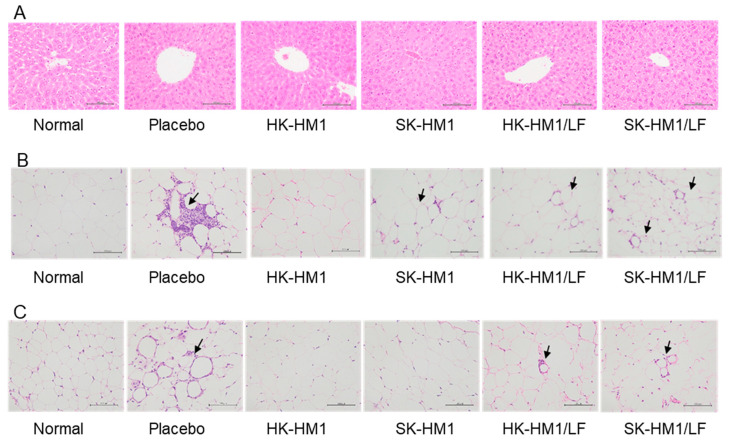
Pathological analysis of liver, epididymal, and perirenal adipose tissues in obese mice following 6 weeks of standard diet and oral administration of inactivated probiotics. (**A**) Liver histopathological sections; (**B**) Epididymal fat histopathological sections; (**C**) Perirenal fat histopathological sections. Pathological lesions are indicated by black arrows in the figure, where black arrows in the fat tissue histopathological sections represent pathological changes related to fat inflammation.

To understand the beneficial effects of dietary control combined with oral probiotic supplementation on tissue pathological changes, we analyzed the extent of tissue lesions in the liver, perirenal fat, and epididymal fat among different experimental groups. Figure 4 illustrates representative results of the aforementioned tissue lesions. As shown in Figure 4A, the livers of mice in both the five experimental groups and the control group exhibited no lesions or inflammatory cell infiltration, and the morphology of their liver cells resembled that of the healthy control group. Since both the placebo group and the probiotic intervention group showed no liver cell lesions, these results support the substantial improvement in liver cell lesions achieved solely through dietary intervention. Additionally, Figure 4B and Figure 4C, respectively, demonstrate inflammatory pathological changes in epididymal fat and perirenal fat among different groups. Notably, we did not observe significant differences in the size and quantity of adipose tissue among different groups (Figure 4B,C); additionally, there were no notable variations in adipose tissue weight between the groups. Conversely, inflammatory pathological changes in adipose tissue were more pronounced (Figure 4B,C). For example, compared to the healthy group, the placebo group still exhibited tissue or inflammatory lesions, while the lesions in the probiotic intervention group were relatively mild. We further calculated the tissue lesion scores and the incidence rates of relevant lesions for all groups (Table 2). First, regarding liver lesions, after 6 weeks of dietary control and any probiotic intervention, all mice in the six groups showed no liver lesions. For epididymal fat lesions, the epididymal fat of healthy mice showed no observed lesions, while in the placebo group, four mice exhibited Grade 1 lesions (66%). However, after additional oral administration of HK-HM1, SK-HM1, HK-HM1/LF, and SK-HM1/LF to obese mice, the probability of developing lesions decreased to 17%, 0%, 50%, and 50%, respectively, with lesion grading reduced to between 0 and 0.5. Regarding perirenal fat lesions, the perirenal fat of healthy mice also showed no lesions, while in the placebo group, four mice exhibited lesions of approximately Grade 0.85 (66%). However, after additional oral administration of HK-HM1, SK-HM1, HK-HM1/LF, and SK-HM1/LF to obese mice, the probability of developing lesions decreased to 17%, 0%, 33.3%, and 50%, respectively, with lesion grading reduced to between 0 and 0.5. 

In summary, our data reveal that a mere dietary change to a regular diet for 6 weeks is sufficient to restore liver tissue slices to a state similar to that of healthy mice. However, in the group that underwent only a dietary change to a regular diet for 6 weeks (placebo group), up to 66% of mice still exhibited mild lesions in epididymal and perirenal fat (approximately between Grade 0.8 and 1). Nevertheless, oral administration of probiotics in mice could significantly reduce the severity and incidence of these lesions. It is noteworthy that the probiotic SK-HM1 demonstrated the most effective improvement in epididymal and perirenal fat lesions, as mice in this group did not exhibit any lesions. 

### 3.5. Impact of Diverse Probiotic Formulations, Coupled with a Standard Chow Diet, on Serum Biochemical Values in Diet-Induced Obese Mice

After 6 weeks of administering a regular diet and probiotics to obese mice, we evaluated their serum biochemical values (Table 3). First, the placebo group exhibited significantly higher serum total triglyceride and total cholesterol levels compared to normal mice (*p* < 0.05). Consequently, dietary control alone did not lead to a reduction in serum total triglyceride and total cholesterol in these mice. However, mice that received HK-HM1 demonstrated a similar level of serum total triglycerides to that of the normal group (*p* > 0.05). Furthermore, mice that received HK-HM1/LF demonstrated a similar level of serum total cholesterol to that of the normal group (*p* > 0.05). These data support the notion that HK-HM1 and HK-HM1/LF supplements could contribute to the improvement in serum total triglycerides or total cholesterol, respectively. On the other hand, a higher level of HDL-c in the serum is generally considered desirable. The results revealed that the placebo group’s serum HDL-c value was significantly higher than that of normal mice (*p* < 0.05). Additionally, the HDL-c values of HK-HM1 and SK-HM1 were also significantly higher than those of normal mice (*p* < 0.05). However, no statistical difference in HDL-C was observed among the placebo, HK-HM1, SK-HM1, and SK-HM1/LF groups. These findings suggest that the higher serum HDL-c levels may be attributed to the effect of the regular diet. Furthermore, concerning LDL-C, there was no statistical difference in LDL-C levels between the placebo group, the four probiotic groups, and normal mice. Finally, in terms of blood glucose indicators, despite higher glucose levels in both the placebo and the four probiotic intervention groups compared to the normal mouse group, only the blood glucose level in HK-HM1/LF significantly exceeded the values observed in normal mice (*p* < 0.05). Moreover, there was no statistical difference in serum glucose among the placebo, HK-HM1, SK-HM1, HK-LM1/LF, and SK-HM1/LF groups (*p* > 0.05), and all were significantly higher than normal mice values (*p* < 0.05). These data suggest that the administration of any of the four inactivated probiotic formulations did not contribute to further control of the blood glucose level with a regular diet. However, as shown by the results of GTT (Figure 2), the glucose tolerance of the placebo group was already similar to that of normal mice. Additionally, after oral administration of HK-HM1/LF and SK-HM1/LF, the glucose tolerance of obese mice was even better than that of normal mice (*p* < 0.05).

In summary, the combined strategy of a regular diet and HK-HM1 showed a tendency toward reducing serum total triglyceride. Furthermore, the combination of a regular diet and HK-HM1/LF also demonstrated a tendency toward decreasing serum total cholesterol. Additionally, dietary control alone increased serum HDL-c and helped maintain LDL-c levels similar to those of normal mice. 

## 4. Discussion

The effectiveness of dietary strategies, such as calorie restriction, in controlling weight gain and metabolic disorders is well established [39,40,41]. However, adherence to such regimens can be challenging, necessitating exploration of alternative approaches. The present study explores the potential metabolic improvements associated with a conventional diet, characterized by standard caloric intake, when supplemented with inactivated probiotics. Specifically, we sought to understand whether this approach could yield beneficial effects on various metabolic indicators without strict calorie reduction. Our results indicated that the current inactivated probiotic formulation provided limited assistance in weight reduction. Notably, the majority of probiotics currently utilized, particularly those employed in sectors such as aquaculture, livestock farming, or economic animal industries, generally exhibit growth-promoting effects, enhancing body weight, improving feed efficiency, and bolstering host immunity against intestinal infections while increasing defense capabilities against specific diseases [42,43,44]. In fact, the probiotic effect on body weight was found to be species- and strain-specific, as highlighted in a recent systematic review. For instance, particular strains may contribute to weight reduction, while other strains might exhibit anti-obesity effects [45]. The limited impact of our probiotics on the body weight of obese mice might also possibly be attributed to the use of inactivated probiotics here. These inactivated probiotics have lost their ability to colonize the gut with live bacteria, potentially resulting in a weaker effect on improving gut microbiota. Supporting this notion, our recent publication revealed that mice receiving live LAB/LF probiotics while being induced into obesity with a high-fat diet consistently exhibited significantly higher body weights than placebo mice throughout most of the induction period. Conversely, mice administered inactivated LAB/LF probiotics showed a significant reduction in body weight gains compared to mice receiving the placebo and live LAB/LF probiotics [34]. While these experiments utilized a mixture of multiple probiotic strains, the results indicated that the oral administration of live and inactivated probiotic formulations plays distinct roles in influencing weight gain. Collectively, concerning the probiotic strains we employed, we believe that the impact of diet on body weight outweighs the simultaneous use of inactivated probiotics. Nevertheless, the current inactivated probiotics we used do not contribute to additional weight gain in obese mice.

In this study, we confirmed that mice subjected to a high-fat diet for 13 weeks not only experienced a weight gain exceeding 40% but also exhibited a decline in glucose tolerance and insulin sensitivity compared to normal mice, aligning with observations reported by previous studies [46,47]. These outcomes substantiate the successful establishment of an obese mouse or non-alcoholic fatty liver disease (NAFLD) mouse model. Within the established obese mouse model, transitioning to a regular diet (standard caloric intake) for 6 weeks not only sustained weight reduction but also improved glucose tolerance and insulin sensitivity, restoring them to levels comparable to normal mice. Importantly, it is noteworthy that mice orally administered with HK-HM1/LF and SK-HM1/LF exhibited glucose tolerance even superior to the normal mice group. Our data also support the idea that orally administering SK-HM1/LF, but not HK-HM1/LF, to mice can further enhance glucose tolerance compared to the placebo control. Notably, the distinction between HK-HM1/LF and SK-HM1/LF lies in the inactivation process of HM1/LF, with HK-HM1/LF undergoing autoclaving. This heating process could disrupt the structure and denature LF partially. We posit that LF in SK-HM1/LF remains active during sonication (in an ice bath) in the inactivation procedure, contributing to the observed improvement in glucose tolerance. Overall, these findings support the potential roles of LF in improving glucose tolerance. To support the advantageous effects of LF in improving glucose tolerance, some previous studies also suggested that LF potentially facilitates glucose regulation [48,49,50]. Moreover, a previous report discovered the anti-diabetic effectiveness of LF through significant improvement in the baseline hemoglobin A1C, body mass index, and lipid profile of an obese pediatric cohort [51]. Additionally, LF has been shown to improve hepatic insulin resistance and pancreatic dysfunction in high-fat diet and streptozotocin-induced diabetic mice [52]. Furthermore, recombinant human LF has been found to attenuate the progression of hepatic steatosis and hepatocellular death by regulating iron and lipid homeostasis in obese mice [53]. In contrast, our data reveal that adding either of the probiotic formulations did not increase insulin sensitivity. It is known that improved glucose tolerance is mostly attributed to two factors: increased glucose-stimulated insulin secretion or increased insulin sensitivity. Since insulin sensitivity was not improved, as observed in the results of the ITT here, it is crucial to determine whether the administration of SK-HM1/LF could contribute to insulin secretion in our next study. Furthermore, investigating the effects of SK-HM1/LF on iron burden or deposition would also aid in dissecting the potential mechanisms of inactivated probiotics on ITT or GTT. 

We intend to investigate this aspect further in our upcoming study, as our current findings suggest that while HK-HM1/LF and HK-HM1/LF supplementation contributed to improved glucose tolerance, they did not enhance insulin sensitivity. On the other hand, our results confirm that improving insulin sensitivity in obese mice can be achieved solely through dietary control (regular diet; normal calorie intake). While the use of inactivated probiotics did not further enhance insulin sensitivity, the fact that improvement was achieved with a regular diet, and not a calorie-restricted one, is noteworthy. This suggests that transitioning from a high-calorie to a normal-calorie diet can be beneficial for health. 

As indicated by previous studies, obese mice may encounter challenges in reverting to their pre-obesity state, even after weight loss, owing to imbalances in gut microbiota and inflammatory responses in fat tissues, which occurred during the period of obesity [54,55,56]. In alignment with prior findings, while our dietary control facilitated weight loss and improved insulin sensitivity and glucose tolerance, it proved insufficient for fully restoring the incidence rate and pathological scores related to inflammation in perirenal and epididymal fat tissues. Significantly, our results suggest that inflammation in these fat tissues—a consequence of obesity—was partially alleviated by the regular diet alone and further improved by the administration of all inactivated probiotic formulations. Notably, SK-HM1 demonstrated the most substantial reduction in inflammation, restoring it to levels comparable to the normal mice group. Nevertheless, our present study emphasizes the advantages of incorporating regular diets with inactivated probiotic formulations in ameliorating inflammation in fat tissues.

Additionally, we observed that dietary control alone, with a regular diet (normal calorie intake), did not reduce the serum total triglyceride and serum total cholesterol levels in mice. However, it led to an increase in serum HDL-c and helped maintain LDL-c levels similar to those of normal mice. Notably, the utilization of HK-HM1 or HK-HM1/LF showed comparable levels of serum total triglyceride or cholesterol values, respectively, compared to those of normal mice groups. Therefore, the use of HK-HM1 or HK-HM1/LF could provide additional benefits in improving the two aforementioned serum biochemical indices.

The current study has certain limitations. It is acknowledged as a pilot study, and the primary objective was to assess whether combining a regular diet with inactivated probiotics could yield improvements in several metabolic indicators. Consequently, the mechanisms underlying the effects of this strategy on improving glucose tolerance and reducing inflammation in perirenal and epididymal fat tissues have not been thoroughly analyzed yet.

In conclusion, this pilot study demonstrates that the combination of a regular diet with non-viable probiotics, whether expressing LF or not, can provide certain benefits in improving various metabolic disorders. For instance, SK-HM1, as opposed to SK-HM1/LF, significantly mitigates the inflammatory status in the examined tissues. Conversely, SK-HM1/LF, rather than SK-HM1, notably improves glucose tolerance, thereby indicating the potential advantages of LF in this context. Our findings also suggest that the deactivation methods applied to probiotics could contribute to varying efficacies in improving metabolic-related indices. However, further efforts are needed to confirm these findings. Nevertheless, the use of inactivated probiotics expressing LF offers several advantages, including addressing concerns related to the use of genetically modified foods. Additionally, these inactivated probiotics exhibit enhanced tolerance to processing methods, increasing their practical applicability. Further research is warranted to delve deeper into the mechanisms and potential long-term effects of this dietary strategy. Finally, the potential roles of LF must be further examined.

## Figures and Tables

**Table 1:** Body weight values of mice at important time points during the experiment. Results are presented as means ± SDs.

Weight (g)	Normal Control	HFD Groups ^a^				
		Placebo	HK-HM1	SK-HM1	HK-HM1/LF	SK-HM1/LF
0 week	23.24 ± 1.2	24.25 ± 1.49	23.44 ± 1.31	22.65 ± 0.61	22.94 ± 1.18	22.96 ± 0.36
13 weeks	26.98 ± 1.39	41.99 ± 4.24 ^#^	40.02 ± 2.74 ^#^	39.12 ± 3.48 ^#^	39.74 ± 3.17 ^#^	38.56 ± 1.86 ^#^
19 weeks—I	27.09 ± 1.18	32.3 ± 1.29 ^#^	30.92 ± 1.23 ^#^	31.49 ± 1.56 ^#^	30.12 ± 1.34 ^#^*	31.8 ± 1.09 ^#^
19 weeks—II (weight gain)	0.1 ± 0.46	−9.69 ± 3.16 ^#^	−9.1 ± 2.95 ^#^	−7.63 ± 2.16 ^#^	−9.62 ± 2.74 ^#^	−6.76 ± 1.45 ^#^

^a^ Various groups of mice were subjected to different diets and probiotic formulations to assess the potential effects of probiotics under diverse conditions. The healthy control group mice (n = 6) were maintained on a regular diet throughout the 19-week experiment. Additionally, four groups of mice (n = 6 for each group) initially received a high-fat diet for 13 weeks, followed by a switch to a standard chow diet (SCD), along with daily oral administration of different probiotic formulations for 6 weeks (from week 13 to 19). Probiotic strain: HK-HM1: heat-killed *Lactobacillus gasseri* HM1; SK-HM1: sonication-killed HM1; HK-HM1/LF: heat-killed lactoferrin-expressing HM1; SK-HM1/LF: sonication-killed lactoferrin-expressing HM1. ^#^ Statistically significant difference compared to Normal control (*p* < 0.05); * Statistically significant difference compared to placebo + SCD (*p* < 0.05).

**Table 2:** The incidence rate and mean score of histopathological lesions or inflammation in the liver, perirenal, and epididymal fat of diet-induced obese mice supplemented with a standard chow diet, along with oral supplementation of various inactivated probiotic strains for 6 weeks.

		Normal	Standard Chow Diet				
			Placebo	HK-HM1	SK-HM1	HK-HM1/LF	SK-HM1/LF
Liver fatty change or inflammation	Incidence rate ^1^	0/6	0/6	0/6	0/6	0/6	0/6
	Mean score ^2^	0 ± 0 ^2,3^	0 ± 0	0 ± 0	0 ± 0	0 ± 0	0 ± 0
Epididymis fat inflammation, Multifocal	Incidence rate	0/6 ^1^	4/6	1/6	0/6	3/6	3/6
	Mean score	0 ± 0 ^2,3^	1.00 ± 0.89 ^#^	0.17 ± 0.41	0 ± 0 *	0.5 ± 0.55	0.5 ± 0.55
Perirenal fat inflammation, Multifocal	Incidence rate	0/6 ^1^	4/6	1/6	0/6	2/6	3/6
	Mean score	0 ± 0 ^2,3^	0.83 ± 0.75 ^#^	0.17 ± 0.41	0 ± 0 *	0.5 ± 0.84	0.5 ± 0.55

^1^ Incidence rate: this was calculated as the number of affected mice divided by the total number of mice examined. ^2^ Lesion severity was graded on a scale from zero to four, based on the following criteria: 0 = normal; 1 = slight (<10%); 2 = moderate (10–33%); 3 = moderate/severe (33–66%); 4 = severe/high (66–100%); ^#^ Statistically significant difference compared to Normal control (*p* < 0.05); ^3^ The final numerical score was calculated by dividing the sum of the number per grade of affected mice by the total number of examined mice. * Statistically significant difference compared to placebo control (*p* < 0.05).

**Table 3:** Effects of various probiotic formulations in combination with regular diet (standard chow diet) on serum biochemical values in diet-induced obese mice.

	Normal	Standard Chow Diet				
		Placebo	HK-HM1	SK-HM1	HK-HM1/LF	SK-HM1/LF
Serum total triglyceride (mg/dL)	11.4 ± 4.49	29.57 ± 9.62 ^#,a^	24.17 ± 4.03 ^a^	28.08 ± 8.12 ^#,a^	26.1 ± 11.01 ^#,a^	33.65 ± 4.65 ^#,a^
Serum total cholesterol (mg/dL)	61.68 ± 9.45	86.32 ± 12.79 ^#,a^	79.47 ± 5.98 ^#,a^	82.52 ± 4.24 ^#,a^	78.43 ± 14.33 ^a^	79.03 ± 6.77 ^#,a^
Serum HDL-C (mg/dL)	50.27 ± 8.27	66.9 ± 6.39 ^#,a^	66.78 ± 5.46 ^#,a^	68.85 ± 4.37 ^#,a^	64.72 ± 13.59	63.8 ± 5.25 ^a^
Serum LDL-C (mg/dL)	9.68 ± 2.74	13.07 ± 4.64 ^a^	9.83 ± 0.98 ^a^	9.68 ± 2.02 ^a^	10.58 ± 2.57 ^a^	10.85 ± 3.37 ^a^
Serum glucose (mg/dL)	106.47 ± 12.98	157.77 ± 54.87 ^a^	146.33 ± 39.56 ^a^	180.97 ± 45.38 ^a^	201.03 ± 54.31 ^#,a^	155.88 ± 38.66 ^a^

^#^ Statistically significant difference compared to Normal group (*p* < 0.05). ^a^ There was no statistically significant difference among placebo, HK-HM1, SK-HM1, HK-HM1/LF, and SK-HM1/LF mouse groups.

## Data Availability

The original contributions presented in the study are included in the article, further inquiries can be directed to the corresponding author.
